# Effects of Solid-State Stretching on Microstructure Evolution and Physical Properties of Isotactic Polypropylene Sheets

**DOI:** 10.3390/polym11040618

**Published:** 2019-04-03

**Authors:** Huajian Ji, Xulin Zhou, Xin Chen, Haili Zhao, Yu Wang, Huihao Zhu, Xiliang Shan, Jin Sha, Yulu Ma, Linsheng Xie

**Affiliations:** School of Mechanical and Power Engineering, East China University of Science and Technology, Shanghai 200237, China; shxiaoji@163.com (H.J.); 15527377408@163.com (X.Z.); chenxin198905@aliyun.com (X.C.); zhl419wsm@163.com (H.Z.); apirl.1992@outlook.com (Y.W.); zhhxhhy1994@126.com (H.Z.); shanxiliang@hotmail.com (X.S.); sjin@ecust.edu.cn (J.S.); myl@ecust.edu.cn (Y.M.)

**Keywords:** solid-state stretching, iPP, cylindrites, SAXS

## Abstract

The microstructure evolution of an isotactic polypropylene (iPP) sheet during solid-state stretching was studied. The transition of the spherulites—cylindrites was evaluated using in-situ two-dimensional wide-angle and small-angle X-ray scattering methods. The crystallinity of stretched iPP sheets was characterized by differential scanning calorimetry. The crystal morphology was observed by means of scanning electron microscopy. It was found that the differences of crystal microstructure of the iPP sheet depended on the stretching strain, which promoted the orientation of molecular chains. Amorphous molecular chains in the spherulites oriented and formed into a mesophase near the yield point, and the partially ordered mesophase was further stretched to form an oriented cylindrite structure after the yield point. The highest relative content of cylindrites appeared at 15% strain. Notably, as the amorphous phase embedded into the lamellae layer, the crystal size decreased with the increase of strain, which indicated that the crystallinity of the stretched iPP sheet was much higher than that of unstretched iPP sheet. The induced cylindrites structure played a more important role in improving the mechanical properties and heat resistance of iPP sheets. Compared with the unstretched iPP sheets, the tensile strength increased by 28%, the notch impact toughness significantly increased by 78%, and the vicat softening point increased from 104 to 112 °C.

## 1. Introduction

Due to its versatile properties, iPP has been widely used to manufacture various products, such as pipes, containers, and automotive interior material [1,2,3]. The tensile strength of iPP is excellent, but impact strength is poor. It is difficult to enhance both tensile and impact properties [4]. As a type of semicrystalline polymer, iPP has two main crystal forms including monoclinic α-iPP and trigonal β-iPP [5,6]. The α-Spherulites of iPP lead to higher modulus and tensile strength but poor toughness and a low heat distortion temperature. The β-Cylindrites of iPP are beneficial for improving the elongation at break and impact toughness [7]. In general, the microstructure of iPP is a thermodynamically stable α-spherulite structure during conventional polymer processing [8,9]. By controlling the crystal structure and morphology, the mechanical properties of iPP can be modified.

The cylindrite structure of iPP can be induced by applying a shear or stretch [10], temperature gradient [11], and β-nucleating agent [12,13], etc. Molecular chains of iPP become extended and slip under stress stretching [14,15]. Fengyuan Yu [16,17] studied the crystallization kinetics of iPP under shear. Their results indicated that shear treatments have a positive effect on the formation of nucleation, and the induction time for crystallization was more sensitive to the increase in the shear rate. Mykhaylyk et al. [18] performed a systematic study of the iPP microstructure and found that oriented cylindrite nucleation appeared when the shear rate exceeded the inverse of the relaxation response time of the longest molecular chain, which indicated that the presence of oriented nucleation was essential for obtaining the cylindrite structure of iPP. Bin Zhang [19] researched the influence of shear stress at the wall of a capillary on iPP cylindrites. The results indicated that the cylindrite component increased as the shear stress increased.

The solid-state stretching also contributes to the slip and rearrangement of the crystal lamellae, ultimately affecting the crystallinity and crystal size of iPP. Many experts have studied the orientation and phase transformation of iPP film during stretching using wide-angle and small-angle X-ray scattering [20,21]. The stretching temperature and strain affected the microstructure of iPP film [22]. Yingguo Zhou et al. [23] studied the effect of the stretching ratio on the oriented structure of iPP film; the results indicated that a spherulitic structure was observed at low draw ratios, and as the draw ratio increased, the spherulites were broken and formed a cylindrite structure along the stretching direction. Strobl [24] studied the elastic modulus and yield stress from the true stress-strain curve of an iPP film. The results revealed that a lamella slippage phenomenon occurred at a critical stretching strain, which indicated that the crystal of the iPP film can be destroyed and recrystallized.

Extensive research has been focused on inducing crystal formation in iPP films, but limited attention has been paid to stretching strain-induced crystal formation in iPP sheets. There are also few studies on the crystal deformation mechanism below the melting point of iPP sheets. Unfortunately, toughness is usually improved at the expense of tensile strength. It is challenging to achieve simultaneous reinforcement and toughening of iPP sheets. Therefore, the morphology and microstructure evolution of the iPP sheet with stretching-induced crystal would be investigated in this study. Self-enhanced purpose was achieved through controlling microstructure of iPP sheets during solid-state stretching. It is a green and environmentally friendly processing method without using the small molecule additives.

## 2. Materials and Methods

### 2.1. Materials and Sample Preparation

Commercial-grade iPP (1100N) was provided by Shenhua Group Corporation Ltd., China, with a melt flow rate (MFR) of 12.8 g/10min (230 °C/2.16 kg). The melting temperature was 165 °C, and the crystallization temperature was 110 °C.

The pelletized iPP granules were melted and injection molded into dog bone-shaped samples (gauge length 50 mm, width 13.2 mm and thickness 3.2 mm). A conventional injection molding machine (COSMOS 80, Ge Lan Manufacture Co., Ltd., Wuxi, China) was used. The temperature from barrel to jet was set to 180, 190, 200, and 210 °C, and the mold temperature was 50 °C. The dog bone-shaped iPP sheet specimens were then stretched by the experimental protocol as follows: (1) iPP sheets were annealed at 110 °C for 5 h to remove residual stress; (2) the high temperature test chamber with a tensile testing machine was heated quickly from room temperature to testing temperature (as 100, 110, 120, 130, 140 °C) with a heating rate of 50 °C/min; (3) iPP sheets were equilibrated for 15 min in the high temperature test chamber and then stretched to the set strain value (as 5%, 10%, 15%, 17%, 20%) at the constant stretching rate (as 1, 3, 5, 7, 10 mm·min^−1^) by stretching fixture; (4) the stretched iPP sheets were maintained for 30 min at stretching temperature and cooled to room temperature at 50 °C/min.

### 2.2. Characterizations

The X-ray Diffraction (XRD) experiments of isotactic polypropylene (iPP) sheets (length 10 mm, width 10 mm and thickness 3 mm) were performed on a D/max 2550VB/PC X-ray diffractometer (Rigaku, Tokyo, Japan) using incident Cu Kα radiation with a wavelength of 1.54 Å at a voltage of 40 kV and a current of 100 mA. The angular range of the wide-angle X-ray diffraction was 3° to 50°, and the step width 2θ was equal to 0.02°. The relative content of cylindrites *K_β_* was determined using the Turner-Jones method [25] as shown in Equation (1):

(1) $$K_{\beta }=\frac{H_{300}}{H_{300}+H_{110}+H_{040}+H_{130}}\times 100\%$$

in which *H*_300_ is the intensity of the *β* peak at 2θ = 16.1° and *H*_100_, *H*_040_ and *H*_130_ are the intensities of the three strong α peaks located at 2θ = 14.2°, 16.6° and 18.5°, respectively.

In-situ two-dimensional wide-angle and small-angle X-ray scattering (2D-WAXS/SAXS) experiments were carried out using a Nano star-u (Bruker AXS Inc., Karlsruhe, Germany) with a Cu Kα radiation source (λ = 0.154 nm). The generator was operated at 50 kV and 600 μA. The two-dimensional scattering patterns were recorded every 60 s by a CCD X-ray detector system in transmission mode (Rayonix, Evanston, IL, USA). The backgrounds of all the WAXS and SAXS patterns had been extracted. CCD was set at 120 and 1070 mm sample-detector distance in the direction of the beam for WAXS and SAXS data collections, respectively. The SAXS intensity was collected with a two-dimensional detector (Vantec-2000, Bruker AXS Inc., Karlsruhe, German). All the data were treated with the software “Fit2D” (European Synchrotron Radiation Facility, Grenoble, France) [3]. In 2D-SAXS patterns, the scattering vectors along and perpendicular to the stretching direction were defined as q_1_ and q_2_, respectively. A one-dimensional intensity distribution was obtained by integrating the intensity at each q_1_ for a certain range of q_2_ as shown in Equation (2):

(2) $$I(q_{1})=\int_{q_{2,a}}^{q_{2,b}}I(q_{1},q_{2})dq_{2}$$

in which *q*_2,*a*_ and *q*_2,*b*_ were selected in such a way that the scattering peak covers the range of the integration.

The thickness of the amorphous and crystalline regions measured along the stretching direction could be derived from the one-dimensional correlation function *K*(*z*) as shown in Equation (3):

(3) $$K(z)=\frac{\int_{0}^{\infty }I(q_{1})\cos(q_{1}z)dq_{1}}{\int_{0}^{\infty }I(q_{1})dq_{1}}$$

in which *z* denoted the stretching direction [26].

To investigate the crystal morphology more thoroughly, the sample surfaces (6 × 6 mm^2^) were etched with a solution containing potassium permanganate in a sulfuric acid and phosphoric acid mixture for 4 h, which mainly removed the amorphous part of the sample. This step made it possible to view the crystal lamellae using a scanning electron microscope (S3400, Tokyo, Hitach, Japan), which had an accelerating voltage of 15 kV. All surfaces were sputtered with gold prior to the test.

The crystallization temperatures and degree of crystallinity of the samples were determined using a TA-Q100 instrument, TA Instruments, New Castle, PA, USA. The experiments were carried out in a temperature range of 20 to 200 °C under a nitrogen atmosphere. 5–7 mg iPP was heated at a rate of 10 °C·min^−1^. Although the DSC curves could exhibit α-and β-fusion peaks, the standard fusion heat of the iPP β-form was minor. The total crystallinity was replaced by the degree of the iPP α-form, as shown in Equation (4):

(4) $$X_{c}=\frac{\Delta H_{m}}{\Delta H_{m}^{0}}\times 100\%$$

in which Δ*H_m_* is the calibrated melting enthalpy of iPP and Δ*H_m_*^0^ is the melting enthalpy of 100% crystalline iPP (207 J·g^−1^) [8].

According to ASTM D1525-09, the softening temperatures were tested using a 1302-B machine (MTS, Eden Prairie, MN, USA). The temperature at which the specimen was penetrated to a depth of 1 mm at a heating rate of 120 °C·h^−1^ by applying 50 N was the softening point.

The tensile strength and flexural properties were tested using an RGM-2020 Universal Testing Machine (Shenzhen Reger Instrument, Shenzhen, China). The notched impact strength was measured using a PTM1100-B1 Impact Testing Machine (SUNS, Shenzhen, China). The sizes of iPP sheets were cut according to ASTM D638, ASTM D790, ASTM D256, respectively. At least five samples were tested in each experiment. All the experiments were carried out at room temperature.

All the values were expressed as mean ± standard deviation (SD). Statistical differences were determined by the analysis of One-Way analysis of variance (ANOVA) and differences were considered statistically significant at *p* < 0.05.

## 3. Results and Discussion

### 3.1. X-ray Diffraction (XRD)

**Figure 1:**
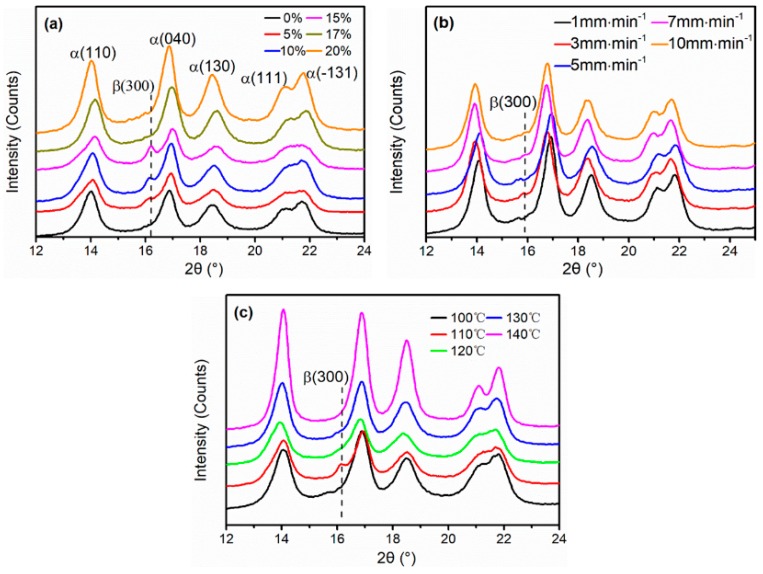
X-ray diffraction (XRD) curves of stretched Isotactic polypropylene (iPP) sheets: (**a**) different stretching strains at 3 mm·min^−1^ with 110 °C; (**b**) different stretching rates under 15% strain with 110 °C; and (**c**) different stretching temperatures at 3 mm·min^−1^ under 15% strain.

The X-ray Diffraction (XRD) curves of the iPP sheets under different stretching conditions are shown in Figure 1. In comparison to the unstretched iPP sheet, the diffraction intensities of the stretched iPP sheet increased, and the full width at half maximum decreased, which indicated that the crystallinity of the iPP sheet increased. In addition, spherulites dominated the crystal region of iPP sheet. This is the most common configuration of the α-crystal in the iPP homopolymer [27]. The XRD diffraction curves of iPP sheets under different stretching strains at 3 mm·min^−1^ with 110 °C are shown in Figure 1a. The relative content of cylindrites *K_β_* obtained from Figure 1a XRD diffraction curves, as calculated by Equation (1), are provided in Table 1. The diffraction angles of 14.2°, 16.6°, 18.5°, 21.0°, and 21.8° corresponded to the (110), (040), (130), (111), and (−131) crystal planes of the α crystal, respectively. The (300) crystal plane at 16.0° corresponded to the β crystal plane [28,29]. In Figure 1a, as the stretching strain gradually increased to 15%, the intensity of the diffraction peak of β (300) increased. *K_β_* increased from 0.001% to 0.964%. In contrast, when the stretching strain was 15% to 20%, *K_β_* decreased from 0.964% to 0.081%. It was explained that the molecular chains in the lamellae underwent stress relaxation under larger strain, and the cylindrites lamellae converted into spherulites lamellae. This phenomenon consisted of the recrystallization of iPP molecular chains under stress relaxation [30].

The X-ray Diffraction (XRD) curves of stretched iPP sheets at different stretching rates under 15% strain with 110 °C are shown in Figure 1b. The diffraction peak intensities did not significantly increase as the stretching rate increased, indicating that the stretching rate had minimal effect on the crystal rate and crystallinity of the iPP sheets. However, the characteristic diffraction peak (300) of the cylindrites at 16.1° appeared when the stretching rate was 3 mm·min^−1^, which indicated that partially anisotropic cylindrites existed in addition to isotropic spherulites in the iPP crystal phase. When the stretching rate was slower (less than 3 mm·min^−1^), the oriented molecular chains had sufficient relaxation time [16], resulting in that the orientation degree of the molecular chains between the spherulites lamellae was low. Therefore, lamellae did not result in a phase transition. When the stretching rate was faster (exceeded 3 mm·min^−1^), the partially oriented molecular chains could quickly recover to amorphous state due to the shorter extension time. There was no obvious crystal diffraction peak β(300) was observed. When stretching rate was 3 mm·min^−1^, the extension and relaxation time of the molecular chains were compatible, and the orientation molecular chains promoted the transition of the spherulites—cylindrites in iPP matrix. In Table 2, the *K_β_* value was the highest at a rate of 3 mm·min^−1^. Therefore, to prepare a stable oriented structure, the optimum value of the stretching rate was 3 mm·min^−1^.

The X-ray Diffraction (XRD) curves of the stretched iPP sheets with different temperatures at 3 mm·min^−1^ under 15% are shown in Figure 1c. The β (300) diffraction peak of the iPP sheets appeared at the crystallization temperature (110 °C). As the stretching temperature increased from 120 °C to 140 °C, the intensities of the α crystal diffraction peaks gradually increased, but the diffraction intensity of the β (300) peak nearly disappeared. Similarly, the β (300) peak also could not founded with the 110 °C stretching temperature in Figure 1c. When the iPP sheets were stretched at higher temperatures, the extended amorphous molecular chains promoted iPP sheet crystallization. However, once the spherulites lamellae occurred slip and formed a thermodynamic metastable mesophase, which would retransform into a stable spherulite structure due to the strong mobility of molecular chains at high temperature. When the iPP sheets were stretched below the crystallization temperature, the β (300) diffraction peak was difficult to be observed in Figure 1c due to the movement of the molecular chains in the amorphous region was weak. Notably, the α-spherulites lamellae were hardly stretched and slipped.

Isotactic polypropylene (iPP) is a highly crystalline polymer. When the iPP sheets were stretched near crystallization temperature (110 °C), the spherulites lamellae could not relax in a short time owing to the restricted movement of the molecular chains. Lamellae structure slipped and formed a cyslindrites nucleus, and the oriented molecular chains grew on it. From Table 3. it could be founded that *K_β_* was nearly zero with the increased stretching temperature. However, it reached the highest at 110 °C. In summary, when the iPP sheet was stretched near the crystallization temperature, the iPP crystal phase exhibited both oriented molecular chains and limited cylindrite structures. In addition, the stretching strain had a greater effect on the microstructure of the iPP sheets than the stretching rate under solid-state condition. 15% strain was the optimum value for inducing stable cylindrites. The stretching strain can affect the movement of the molecular chains and induce lamellae slippage in the crystal structure of the iPP sheet.

### 3.2. Differential Scanning Calorimetry (DSC)

**Figure 2:**
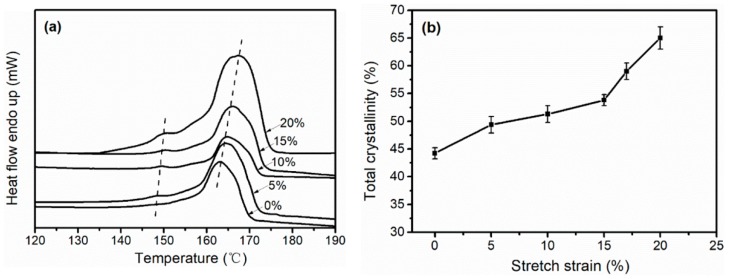
Differential scanning calorimetry (DSC) melt curves (**a**) and total crystallinity (**b**) of stretched iPP sheets under different stretching strains at 3 mm·min^−1^ with 110 °C.

The (Differential Scanning Calorimetry) DSC curves and total crystallinity of the stretched iPP sheets under different stretching strains at 3 mm·min^−1^ with 110 °C are shown in Figure 2. In Figure 2a, all the curves for the iPP sheets contained two peaks except for the curve with the unstretched iPP sheet. The minor peak represented the melting peak of the cylindrites, and this peak became more distinct as the stretching strain increased, implying that the cylindrite content gradually increased [2]. This result is consistent with that shown in Figure 1a. The large peak represented the melting peak of the spherulites, which increased with the increasing of stretching strains. The total crystallinity values of the stretched iPP sheets according to equation 4 are shown in Figure 2b. The total crystallinity increased by 27.9% as the stretching strain increased from 0% to 20%.

In the stage of elastic deformation (<5% strain), the orientation of the amorphous molecular chains accelerated the three-dimensional ordered structure, which made crystallization easier. In the stage of yield (5–15% strain), the transition of spherulites–cylindrites in iPP matrix expensed the external work, so the crystallinity increased slowly. Furthermore, in the stage of plastic deformation (15–20% strain), the slippage of lamellae reduced the free energy of crystallization, which promoted the crystal growth and made crystallization more perfect, eventually leading to the increase in melting point. Solid-state stretching could induce the traditional spherulites to transform into cylindrites structure, and increase the melting point of the iPP sheet, which enhances the thermal behavior of the sheet.

### 3.3. 2D-Wide-Angle X-Ray Scattering (WAXS)/Small-Angle X-Ray Scattering (SAXS)

**Figure 3:**
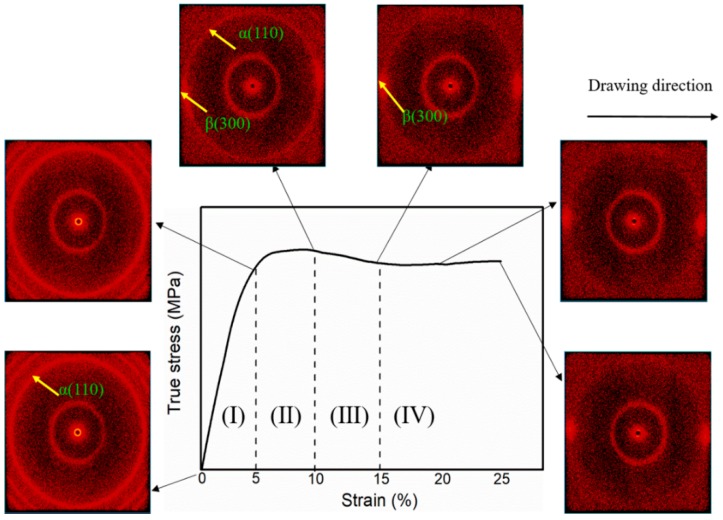
Two-dimensional wide-angle X-ray scattering patterns of in-situ solid-state stretching for the iPP sheet at 3 mm·min^−1^ with 110 °C.

To further elucidate the microstructure evolution of the iPP sheets as a function of stretching strain, in situ two-dimensional wide-angle X-ray scattering (2D-WAXS) profiles during stretching at 3 mm·min^−1^ with 110 °C were recorded, and they are shown in Figure 3. The true stress-strain curve of the iPP sheets is also shown in Figure 3. When the stretching strain was less than 5% (I) prior to the yield point, a typical spherulite diffraction circular ring for α (110) was observed. When the stretching strain reached 10% (II) near the yield point, a weak diffraction arc for β (300) was observed along the stretching direction. Then, the diffraction arc for β (300) became more obvious as the stretching strain increased from 10–15% (III). which indicated that the content of cylindrites gradually increased. However, when the stretching strain increased from 15% to 25% (Ⅳ), the diffraction intensity of arc β (300) was not significantly enhanced, which implied that the content of cylindrites reached a maximum at a stretching strain of 15%.

Prior to the yield point, when stretching strain was less than 5%, the extended amorphous molecular chains could recover quickly, the spherulites lamellae did not occur slippage. The characteristic diffraction peak (300) of β cylindrites was not observed from the 2D-WAXS pattern. When stretching strain was close to the yield point, the diffraction peak (300) of β cylindrites appeared, which indicated that the spherulites lamellae occurred slippage. Mesophase structures between the spherulite lamellae were induced by solid-state stretching strain [31,32]. The thermodynamic metastable mesophase which depended on stretching strain further converted into the stable cylindrites by external work. Interestingly, the content of cylindrites did not increase significantly with the increase of stretching strain as shown in Figure 3. Metastable mesophase structure was restored to the spherical lamellae structure due to the stress relaxation of the molecular chain under external work. Therefore, the formation of the mesophase is a dynamic equilibrium between molecular chain relaxation and lamella slippage. This is why it is difficult for the cylindrites to exist in PP homopolymers during conventional processing.

**Figure 4:**
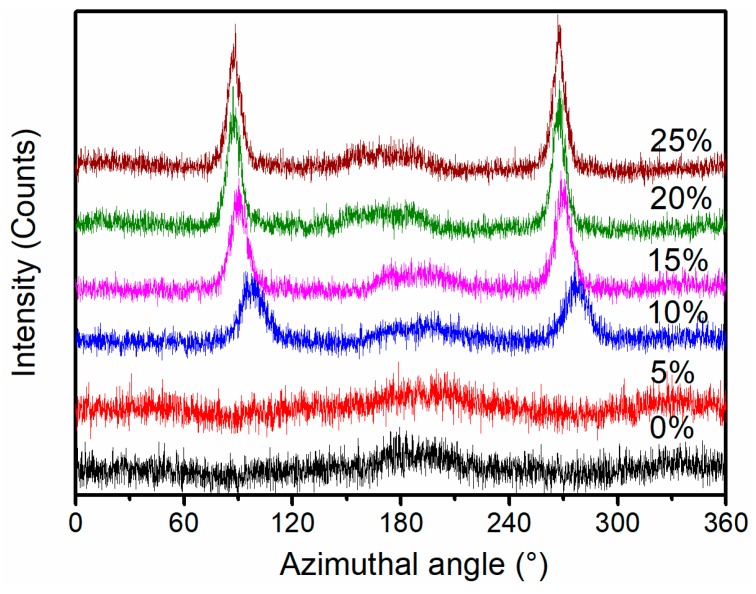
One-dimensional wide-angle X-ray scattering integral intensity distribution of iPP sheet under different stretching strains at 3 mm·min^−1^ with 110 °C.

One-dimensional wide-angle X-ray scattering (1D-WAXS) integral intensity curves obtained from the 2D-WAXS data are shown in Figure 4; 90° and 270° were the characteristic azimuthal angles of cylindrites [33]. When the stretching strain was below 5%, diffraction peaks were not observed at azimuthal angles of 90° and 270°, which indicated that oriented cylindrites did not exist in the iPP sheet. When the stretching strain increased to 10%, diffraction peaks at azimuthal angles of 90° and 270° appeared, indicating that the stable oriented cylindrites existed. The intensities of the azimuthal angles at 90° and 270° became stronger as the stretching strain increased to 15%, which consisted of the results of Figure 3. However, the intensities of the azimuthal angles at 90° and 270° did not continually enhance when the stretching strain increased from 15% to 25%, implying that the content of cylindrites reached the highest under 15% strain.

**Figure 5:**
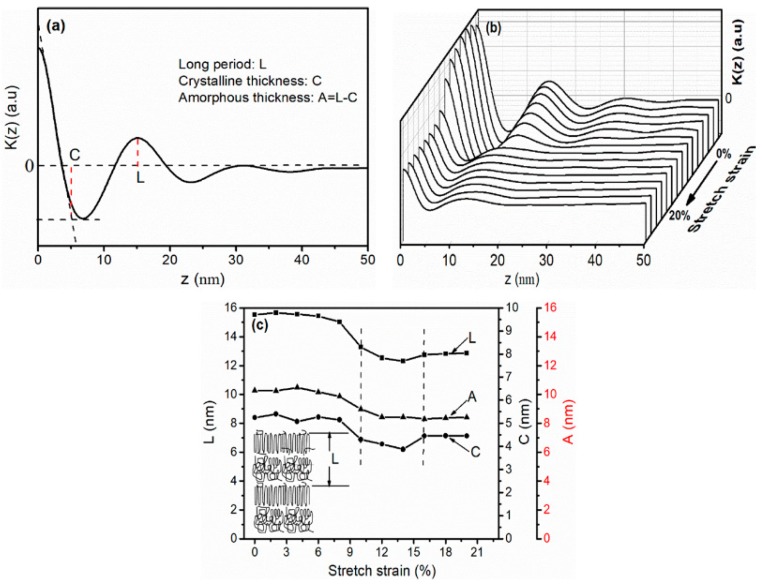
One-dimensional correlation function K(z) of in-situ solid-state stretching for the iPP sheet at 3 mm·min^−1^ with 110 °C: (**a**) 0% strain; (**b**) 0%–20% strain; and (**c**) The crystal sizes of in-situ solid-state stretching for the iPP sheet at 3 mm·min^−1^ with 110 °C.

It can be seen from the previous studies that solid-state stretching could modify the crystal structure and increase the crystallinity of iPP sheet. In order to explain in detail the effect of solid-state stretching on the crystal structure size, one-dimensional correlation function *K*(*z*) derived from Equations (2) and (3) of the stretched iPP sheet under different stretching strains are shown in Figure 5. Various parameters were extracted (as indicated in Figure 5a) from the correlation function [34,35]. Long period L corresponded to the thickness of the crystalline layer C plus the amorphous layers in the crystal block. K (z)-z curves of the stretched iPP sheet during in-situ solid-state stretching at 3 mm·min^−1^ with 110 °C are shown in Figure 5b. The quantitative data obtained from Figure 5b are shown in Figure 5c.

When the stretching strain was smaller, the long period L and lamellae thickness C were approximately 15.5 and 5.4 nm, respectively (Figure 5c). In the elastic deformation stage, the original lamellae could not be stretched, and the lamellae thickness C changed slightly. However, the oriented molecular chains along the stretching direction emerged between the neighbouring lamellae. The insertion of an oriented structure counteracted the increase in the average distance between the adjacent lamellae. Therefore, the long period L did not increase significantly. When the stretching strain increased from 10% to 15%, which exceeded the yield point, the long period L and lamellae thickness C decreased to 12.6 and 4.1 nm, respectively. On the one hand, the oriented mesophase partially converted into oriented cylindrites with further plastic deformation, and the amorphous molecular chains were embedded into the lamellae. On the other hand, transformation of the amorphous phase between the lamellae to the mesophase led to a large decrease in the entropy, which could also explain the reduction in the long period. When the stretching strain continued to reach 20%, the long period L and lamellae thickness C were approximately increased to 13.1 and 4.5 nm, respectively. The interface between the lamellae and the ordered molecular chains separated, resulting in a slight increase in the distance between the adjacent lamellae. Mao et al. [36] studied the change of a long period of iPP as a function of stretching strain, where the long period decreased owing to lamellae became densely packed and long period increased because of the separation of lamellae.

Solid-state stretching can reduce the long period of iPP sheet. It also could be seen from Figure 2 that the crystallinity of the iPP sheet increased under the stretching strain. It implied that compared with unstretched iPP sheet, more nucleation sites were formed and the number of crystal increased in the iPP matrix. When the spherulites lamellae were induced in stretching strain under external work, the long period reduced to a certain value, which resulted in the spherulites breaking to oriented cylindrites nucleus. The random molecular chains grew on the surface of cylindrites nucleus and formed stable cylindrites. The increasing of crystals facilitated the transfer of stress and heat in iPP sheet, which is advantageous for the improvement of the mechanical properties and thermal properties of iPP sheet.

### 3.4. Scanning Electron Microscopy (SEM)

**Figure 6:**
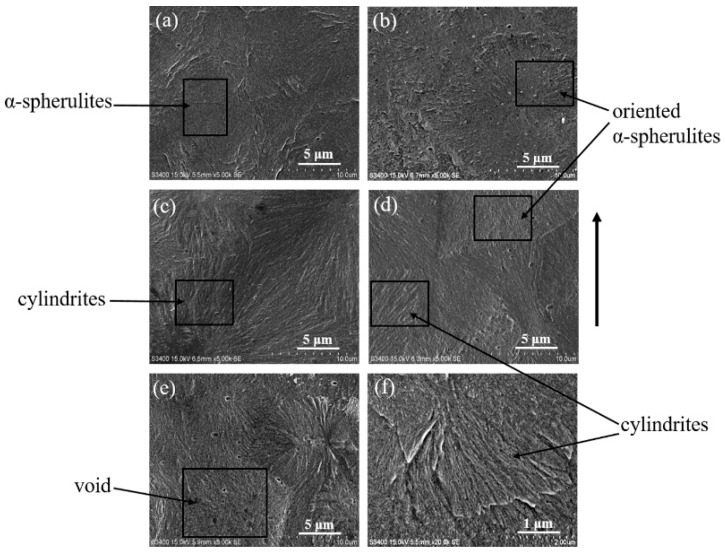
Scanning electron microscopy (SEM) images reflecting the microscopic crystal morphology of stretched iPP sheets under different stretching strains at 3 mm·min^−1^ with 110 °C: (**a**) 0% strain; (**b**) 5% strain; (**c**) 10% strain; (**d**,**f**) 15% strain; (**e**) 20% strain; and (**f**) magnification of (**d**). The arrow indicates the stretching direction.

The (Scanning Electron Microscopy) SEM images that illustrate the crystalline morphology of stretched iPP sheets under different strains are shown in Figure 6. The crystal phase of the stretched iPP sheet under small stretching strains predominantly consisted of rigorous α-spherulites, as shown in Figure 6a,b [37]. When the stretching strain increased from 10% to 15%, a small amount of cylindrites morphology was observed (Figure 6c,d). When the stretching strain was 20%, a few voids were observed besides the cylindrites (Figure 6e). As the stretching strain was small, the molecular chains between lamellae became slightly oriented, amorphous molecular chains were stretched easily, and partial spherulites transformed into oriented α-spherulites. As the stretching strain increased, the molecular chains between the lamellae were stretched to form a mesophase structure, and the crystal phase transition process of the spherulites occurred. In addition, a cylindrite morphology was also observed. However, when the stretching strain was 20%, the interface between the crystal and amorphous phases was poor in the plastic deformation. Phase separation is possible and have resulted in the formation of the previously mentioned voids [38]. The voids caused more stress concentration points and reduced the strength of the iPP sheet. Therefore, a larger strain was not conducive to microstructure development in the iPP sheet.

### 3.5. Cylindrite Structure Transformation Mechanism

When the iPP sheet was stretched at a specific temperature, the free energy of crystallization decreased [17,39], and the molecular chains oriented along the stretching direction. Spherulites lamellae molecular chains did not relax in a short period of time due to the restricted movement of the molecular chains at the crystallization temperature. A few spherulites lamellae would be converted into cylindrites. The stretching rate also affected the crystal structure. Only when the relaxation time and extension time of the molecular chain were compatible, the molecular chains between the lamellae could form a stable mesophase structure. The polymer subjected to solid-state stretch during processing, and the movement mode of molecular chains before and after the yield point are different. According to this study, the effect of the stretching strain on the microstructure was more practical. When the stretching strain was smaller than the yield point, elastic deformation existed in the amorphous phase, and this deformation was a reversible process that could be quickly reversed. When the stretching strain exceeded the yield point, the amorphous molecular chains between the lamellae underwent plastic deformation and orientation.

**Figure 7:**
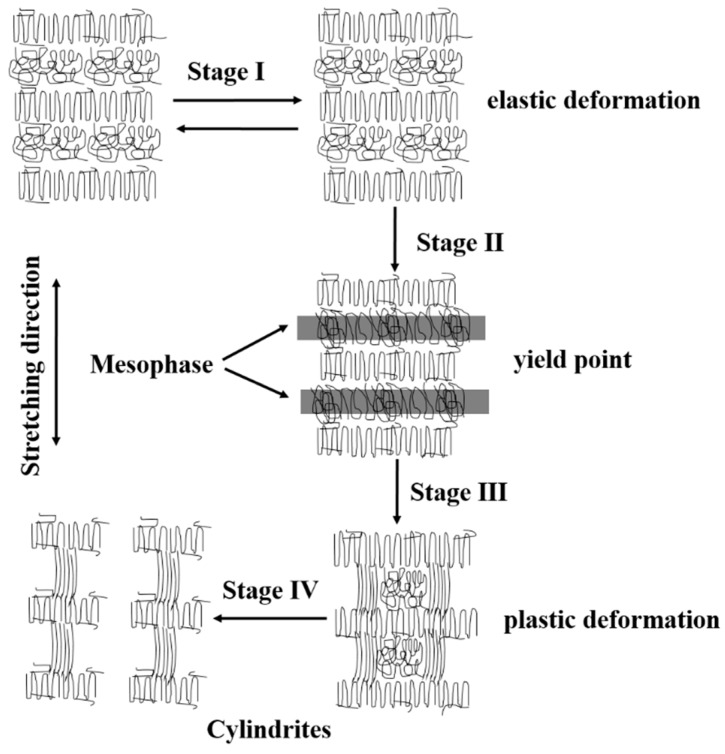
A schematic diagram reflecting the transition of the spherulites—cylindrites of stretched iPP sheets with the crystallization temperature.

Based on the experimental results, a schematic representation of the mechanism is shown in Figure 7 to illustrate the deformation of cylindrites during uniaxial tension. The transition of spherulites—cylindrites of stretched iPP sheet involved four stages. When the iPP sheet was stretched under a small strain, the spherulite lamellae did not slip or break because of elastic recover. However, a small amount of spherulites was stretched to form oriented spherulites (Stage I). When the stretching strain increased to the yield stage, the conformation of the amorphous molecular chains between adjacent lamellae changed, which resulted in the generation of mesophase structure due to the restricted movement of the molecular chains (Stage II). When the stretching strain increased in the yield stage, the oriented mesophase embedded into folded chain lamellae and formed cylindrites precursor (Stage III). When the stretching strain further increased, the cylindrite precursor would be broken into stable cylindrite (Stage Ⅳ).

### 3.6. Mechanical and Thermal Properties

**Figure 8:**
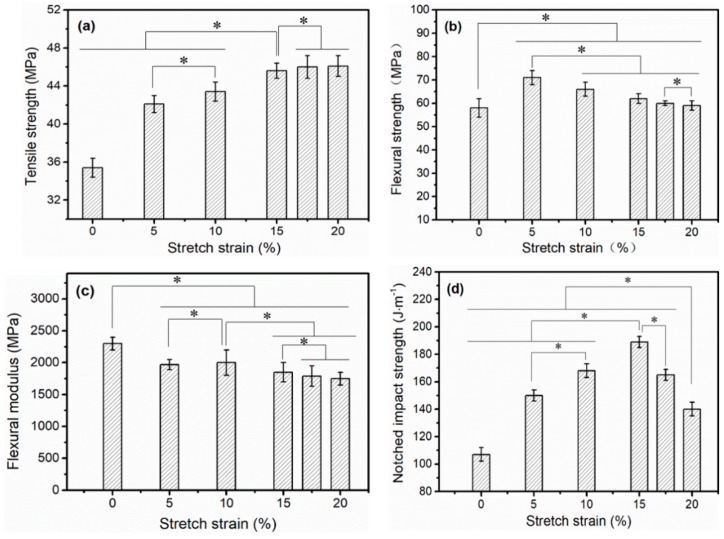
Tensile strength (**a**); flexural strength (**b**); flexural modulus (**c**); and notched impact strength (**d**), of stretched iPP sheets under different stretching strains at 3 mm·min^−1^ with 110 °C. (* *p* < 0.05).

Structure determines performance, and solid-state stretching could induce crystal structure transformation, which would also improve the mechanical properties of iPP sheets. Sheng et al. [3] studied the nucleating agent-reinforced iPP sheet and found that the toughness was significantly improved but the tensile strength was hardly improved. It was difficult to enhance both tensile and impact strength. The mechanical strength of stretched iPP sheets under different stretching strains is shown in Figure 8. The relationship between the tensile strength and solid-state stretching strain is shown in Figure 8a. When the stretching strain increased from 0% to 15%, the tensile strength increased by 28%. Then, the tensile strength did not markedly increase as the stretching strain reached 20%. Studies have shown that the higher the crystallinity, the higher the tensile strength [40]. When the stretching strain was smaller than 15%, the orientation of the amorphous molecular chains led to an increase in the crystallinity, which improved the tensile strength of the iPP sheet. When the stretching strain gradually increased from 15% to 20%, voids were observed in the microstructure of the iPP sheet (Figure 6e), which formed stress concentration points to reduce the tensile properties of iPP sheets. However, the result of Figure 2b showed that the crystallinity rapidly increased. Therefore, the tensile strength did not decrease or increase significantly.

The flexural strength and modulus as a function of the stretching strain are shown in Figure 8b,c. In comparison to the unstretched iPP sheet, the flexural strength of the stretched iPP sheets increased significantly, and the flexural strength had a maximum value of 71.1 MPa at a stretching strain of 5%. Subsequently, as the stretching strain increased, the flexural strength gradually decreased. When the stretching strain was smaller than 5%, the orientation of the amorphous molecular chains perpendicular stretching direction, which improved the flexural strength of the iPP sheet perpendicular stretching direction. When the stretching strain exceeded 5%, the orientation of molecular chains along the stretching direction increased the crystallinity, but the molecular chains perpendicular to the stretching direction recover the random state owing to stress relaxation. The flexural strength of perpendicular to stretching direction had a reduction with the increasing of stretching strain. In addition, the flexural modulus changed minimally as the stretching strain increased. The stretching strain mainly affected the flexural strength of the material and had a limited effect on the modulus.

The relationship between the notched impact strength and stretching strains is shown in Figure 8d. When the stretching strain increased from 0% to 15%, the notched impact strength rapidly increased to a maximum of 189 J·m^−1^. When the stretching strain increased from 15% to 20%, the notched impact strength decreased from 189 to 140 J·m^−1^. Cylindrites structures could absorb more energy owing to β-α phase transition under stress loading [4]. Therefore, the notched impact strength increased with the increasing of the cylindrites content, and reached the highest under 15% stretching strain. When the stretching strain reached 20%, the impact energy was not absorbed effectively due to the presence of voids resulting in the reduction of notched impact strength.

**Figure 9:**
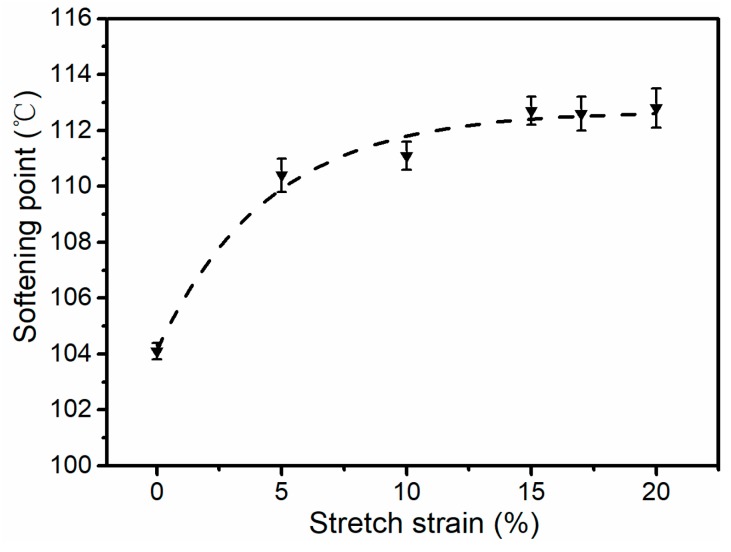
Softening point temperatures of stretched iPP sheets under different stretching strains at 3 mm·min^−1^ with 110 °C.

Vicat softening point temperatures of the stretched iPP sheets under different stretching strains are shown in Figure 9. It could be founded that the softening point temperature increased from 104 to 112 °C as the stretching strain increased from 0% to 15%, and it had a little change with the stretching strain increasing from 15–20%. When the stretched iPP sheets were heated, the cylindrites absorbed energy and gradually converted into a thermodynamically more stable spherulites structure. That is, the softening point temperature increased as the relative content of cylindrites *K_β_* increased. The *K_β_* reached the highest at 15% strain according to Table 1. However, the increasing of the number of crystal in stretched iPP sheet was beneficial to the absorption and transfer of energy, so that the softening point temperature did not decrease significantly after the stretching strain exceeded 15%. Here, a plateau region was observed in Figure 9 when the stretching strain increased from 15% to 20%.

It has been found that applying a relatively small stretching strain in the vicinity of the crystallization temperature in the iPP sheet can induce crystal transition. The modified crystal structure can enhance both tensile and impact strength. Especially, the method of small strain solid-state stretching can be used in the blow molding process of hollow products. The impact and tensile strength could be enhanced by changing the microstructure of iPP, ultimately, save cost and achieve high performance of general materials. That has important guiding significance for the preparation of industrial large-capacity barrel products. Compared to the stretching films, only larger strain (hundreds or even thousand) can induce crystal transition in stretching films.

## 4. Conclusions

This study examined the solid-state stretching strain-induced crystal deformation process of iPP sheets using in-situ two-dimensional wide-angle and small-angle X-ray scattering. Crystal transition and orientation of iPP sheets were evaluated during stretching. Spherulites lamellae were stretched and formed a mesophase precursor near the yield point at the crystallization temperature. Subsequently, the transition of spherulite—cylindrites took place based on the existence of mesophase precursor. The relative content of cylindrites achieved the highest at 15% strain. The long period and lamellae thickness of crystal reduced under solid-state stretching strain, which promoted the mechanical properties and heat resistance of iPP sheets. The mechanical properties of the iPP sheet was optimized at 15% strain, especially the notched impact strength increased by 78% and the vicat softening point increased from 104 to 112 °C. Simultaneously increased tensile strength and toughness by self-reinforcing with low strain in solid-state stretching, which was of great significance in the actual processing.

## Figures and Tables

**Table 1:** The relative content of cylindrites of stretched iPP sheets under different stretching strains at 3 mm·min^−1^ with 110 °C.

ε ^a^ (%)	T ^b^ (°C)	V ^c^ (mm·min^−1^)	*K_β_*^d^ ± SD (%)
0	110	3	0.001 ± 0.001
5	110	3	0.143 ± 0.002
10	110	3	0.354 ± 0.001
15	110	3	0.964 ± 0.003
17	110	3	0.192 ± 0.002
20	110	3	0.081 ± 0.001

^a^ Stretch strain; ^b^ Stretch temperature; ^c^ Stretch rate; ^d^ The relative content of cylindrites *K_β_* calculated by Turner-Jones method [25], standard deviation (SD).

**Table 2:** The relative content of cylindrites of stretched iPP sheets at different stretching rates under 15% strain with 110 °C.

V ^a^ (mm·min^−1^)	T ^b^ (°C)	ε ^c^ (%)	*K_β_*^d^ ± SD (%)
1	110	15	0.002 ± 0.001
3	110	15	0.964 ± 0.003
5	110	15	0.052 ± 0.002
7	110	15	0.063 ± 0.001
10	110	15	0.091 ± 0.002

^a^ Stretch rate; ^b^ Stretch temperature; ^c^ Stretch strain; ^d^ The relative content of cylindrites *K_β_* calculated by Turner-Jones method [25], standard deviation (SD).

**Table 3:** The relative content of cylindrites of stretched iPP sheets with different stretching temperatures at 3 mm·min^−1^ under 15% strain.

T ^a^ (°C)	ε ^b^ (%)	V ^c^ (mm·min^−1^)	*K_β_*^d^ ± SD (%)
100	15	3	0.002 ± 0.001
110	15	3	0.964 ± 0.003
120	15	3	0.001 ± 0.001
130	15	3	0.002 ± 0.001
140	15	3	0.002 ± 0.001

^a^ Stretch temperature; ^b^ Stretch strain; ^c^ Stretch rate; ^d^ The relative content of cylindrites *K_β_* calculated by Turner-Jones method [25], standard deviation (SD).
